# The Effects of Exercise on Plaque Volume and Composition in a Mouse Model of Early and Late Life Atherosclerosis

**DOI:** 10.3389/fcvm.2022.837371

**Published:** 2022-03-28

**Authors:** Kelly M. Stanton, Hongjuan Liu, Vivian Kienzle, Christina Bursill, Shisan Bao, David S. Celermajer

**Affiliations:** ^1^Clinical Research Group, Heart Research Institute, Sydney, NSW, Australia; ^2^Faculty of Medicine and Health, The University of Sydney, Sydney, NSW, Australia; ^3^Discipline of Pathology and School of Medical Science, University of Sydney, Sydney, NSW, Australia; ^4^Vascular Research Centre, South Australian Health and Medical Research Institute, Adelaide, SA, Australia; ^5^Faculty of Health and Medical Science, University of Adelaide, Adelaide, SA, Australia

**Keywords:** exercise, atherosclerosis, cholesterol, *apoE*^−/−^ mice, MMP – matrix metalloproteinase, TIMP – tissue inhibitors of metalloproteinase

## Abstract

**Background:**

Exercise is associated with a less atherogenic lipid profile; however, there is limited research on the effect of exercise on atherosclerotic plaque composition and markers of plaque stability.

**Methods:**

A total of 110 apolipoprotein (*apo*)*E*^−/−^ mice were placed on a chow diet and randomly assigned to control or exercise for a period of 10 weeks, commencing either at 12 weeks of age (the early-stage atherosclerosis, EA group) or at 40 weeks of age (the late-stage atherosclerosis, LA group). At the end of the exercise period, blood was assayed for lipids. Histologic analysis of the aortic sinus was undertaken to assess plaque size and composition that includes macrophage content, monocyte chemoattractant protein (MCP)-1, matrix metalloproteinase-2 (MMP-2), and tissue inhibitors of metalloproteinase 1 and 2 (TIMP-1 and 2).

**Results:**

A total of 103 mice (38 EA, 65 LA) completed the protocol. In the EA group, exercise reduced plasma total cholesterol (TC) (−16%), free cholesterol (−13%), triglyceride (TG) (−35%), and phospholipid (−27%) levels, when compared to sedentary control mice (*p* < 0.01). In the EA group, exercise also significantly reduced plaque stenosis (−25%, *p* < 0.01), and there were higher levels of elastin (3-fold increase, *p* < 0.0001) and collagen (11-fold increase, *p* < 0.0001) in plaques, compared to control mice. There was an increase in plaque MMP-2 content in the exercise group (13% increase, *p* < 0.05) but no significant difference in macrophage or MCP-1 content. In the LA group, exercise reduced plaque stenosis (−18%, *p* < 0.05), but there was no significant difference in plaque composition. There was no difference in macrophage, MCP-1, or MMP-2 content in the LA groups. TIMP-1 was lower with exercise in both the EA and LA groups (−59%, *p* < 0.01 and −51%, *p* < 0.01 respectively); however, there was no difference in TIMP-2 levels.

**Conclusion:**

A 10-week exercise period reduces atherosclerotic plaque stenosis when commenced at both early- and late-stage atherosclerosis. Intervening earlier with exercise had a greater beneficial effect on lipids and plaque composition than when starting exercise at a later disease stage.

## Introduction

Atherosclerosis is a complex disease process, which is characterized by the formation of atherosclerotic plaques due to the accumulation of lipids and lipoprotein particles in the intima of arterial walls, along with endothelial dysfunction, inflammation, and smooth muscle cell (SMC) proliferation (1). Stages of atherosclerosis have been described and the concept of the “vulnerable plaque” is now well established (2). Despite significant advances in medical therapies and interventional techniques, atherosclerosis-related heart disease remains the leading cause of mortality worldwide.

Vulnerable atherosclerotic plaques are characterized by a paucity of SMCs, an accumulation of macrophage-derived foam cells and thin fibrous cap (3). Thinning of the fibrous cap is due to extracellular matrix degradation, which is thought to be due to increased matrix metalloproteinase (MMP) activity or an imbalance between MMP activity and the activity of their endogenous inhibitor (tissue inhibitors of MMPs; TIMPs) (4–6). In addition to increased MMP activity, increased monocyte chemoattractant protein (MCP)-1 activity has been shown to enhance recruitment of monocytes to sites of atherosclerosis and have an important role in atherosclerosis progression and plaque instability.

Exercise is a well-established protective factor for atherosclerotic disease. Large-scale epidemiologic studies have shown that exercise reduces the risk of cardiovascular events (7). The mechanism by which exercise has this effect is not well understood but is thought to be due to improved traditional risk factors that include increased HDL-C levels and function, and anti-inflammatory, anti-oxidant, and antithrombotic effects. Exercise studies are predominantly cross-sectional and look at biomarkers of cardiovascular disease or cardiovascular events, with only very few looking at plaque composition. In particular, a detailed evaluation of the atherosclerosis-related effects of exercise at various stages of plaque development is yet to be performed.

Earlier preclinical studies from our center have demonstrated a differential effect of apolipoprotein A-1 (apoA-1) infusions in early-stage atherosclerosis vs. late-stage plaque (4). This highlights that the interventions targeted at atherosclerosis may have differential effects depending on the stage of atherosclerosis.

This study therefore assessed the effects of exercise on atherosclerotic plaque size and composition in both an early- and late-stage mouse model of atherosclerosis, to determine the effect of exercise on plaque composition and also its effect on early- vs. late-stage disease.

## Materials and Methods

### General Animal Protocol

Male apolipoprotein E-deficient (*apoE*^−/−^) mice on C57Bl/6J background were housed in standard high-top filter cages and fed chow diet *ad libitum* (Specialty Feeds, Glen Forrest, Australia). Blood pressure, weight, and heart rate were measured prior to randomization and at the end of each protocol. Tail cuff plethysmography was used to measure blood pressure and heart rate (IITC Life Science, CA, USA).

This project was approved by the Royal Prince Alfred Hospital Animal Ethics committee (protocol number 2014-011). All animal handling techniques were in line with the approved research protocol.

**Figure 1:**
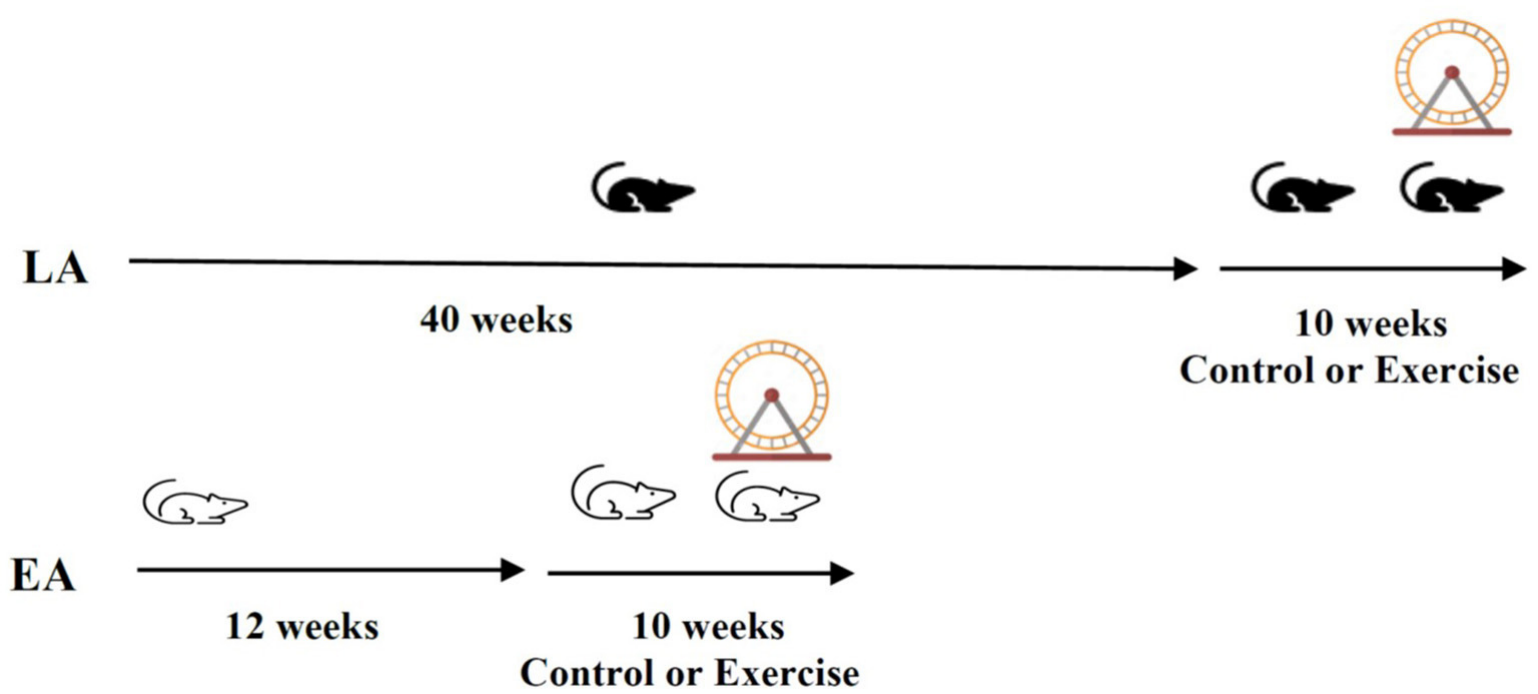
Study protocol: Male *apoE*^−/−^ mice on a chow diet (*n* = 110 were randomly allocated to the late-stage or early-stage atherosclerosis group (LA; *n* = 65, EA; *n* = 38). They were further randomly allocated to control or exercise groups at 12 (EA) or 40 weeks (LA) of age for a further 10 weeks.

One hundred and ten *apoE*^−/−^ mice participated in the study. At 12 weeks of age, they were randomly assigned to either the early- or late-stage atherosclerosis group (Figure 1). The early-stage atherosclerosis group (12 weeks of age) was then further randomly assigned to control or exercise for a period of 10 weeks. A total of thirty-eight mice completed the early-stage atherosclerosis protocol (20 exercise group, 18 controls). The late-stage atherosclerosis group was randomly assigned to control or exercise at 40 weeks of age, also for a period of 10 weeks. A total of sixty-five mice completed the late-stage atherosclerosis protocol (32 exercise group, 33 control).

### Exercise Protocol

The mice assigned to the exercise groups were individually housed, and a running wheel [wireless low-profile mouse running wheel (Med Associates, Vermont, USA, MEDASS ENV-047)] was added to each cage at the start of the exercise intervention (Figure 1). In both exercise groups, mice were exposed to 10 weeks of exercise (running wheel). Mice were able to run voluntarily but were also received daily encouragement. Running data were tracked using Wireless Environmental Sensor (MEDASS, ENV-044E).

Mice in the control groups were individually housed at the same time points as their corresponding exercise group and were exposed to standard environmental enrichment but no running wheel.

### Tissue Collection and Processing

Mice were anesthetized with methoxyflurane (Sigma-Aldrich) followed by right ventricular puncture for blood collection. Blood was centrifuged at 5,000 RPM for 5 min, and plasma was collected and stored at −80°C.

Plasma was analyzed using the chemistry analyzer AU480 (Beckman Coulter Inc., CA, USA) for colorimetric analysis of total cholesterol (TC), free cholesterol (FC), triglyceride (TG) phospholipid (PL), and protein levels.

High-density lipoprotein cholesterol (HDL-C) concentration was determined using a commercially available mouse ELISA kit (Cloud-Clone Corp., TX, USA, SEB006MU-96T). Apolipoprotein B (apoB) concentration was determined using a commercially available apoB mouse ELISA kit (CUSABIO.com, CSB-EL0019 18 MO-96T). All samples were analyzed in duplicate.

### Tissue Embedding and Aortic Sinus Sectioning

The aortic arch and aortic sinus were harvested at sacrifice. Samples were fixed in 4% (w/v) paraformaldehyde (PFA) at 4°C overnight and then 70% (v/v) ethanol for 24 h, as described (8, 9). After tissue fixation by standard techniques, samples were paraffin embedded (9).

The aortic sinus block was orientated with all three aortic leaflets in view. The wax blocks were sectioned at 5 μm, using a microtome and collected on glass slides (Menzel-Glaser, Germany) for histochemical staining and immunohistochemical analysis. Serial sections at 5 μm (15 slides) spanning the aortic sinus were cut and mounted, and 3 sections (S1, S10, and S15) were used for the determination of plaque area. These were stained with hematoxylin and eosin (H&E).

Remaining sections were used to characterize plaque composition. Slides were dried and stored at 4°C. Prior to histochemical and immunohistochemistry stains, sections were deparaffinized and rehydrated according to the standard techniques, as described previously.

### Histochemical and Immunohistochemistry Stains

Slides were stained with H&E and pentachrome Movat's stain for histomorphometric analysis. H&E stain was used to calculate plaque area, volume, and percentage stenosis. Pentachrome Movat's stain was used to assess plaque for elastin and collagen content.

Remaining slides were stained with anti-F4/80 (1:250) (macrophages Abcam, Sydney Australia), anti-MMP-2 (1:400) (Abcam, Sydney Australia), anti-TIMP-1 (1:800) (Abcam, Sydney Australia), anti-TIMP-2 (1:200) (Abcam, Sydney Australia), anti-MCP-1 (1:200) (Abcam, Sydney Australia), and anti-human Smooth Muscle Actin Clone 1A4 (1:300) (DAKO, Sydney Australia) antibodies followed by the appropriate secondary antibodies, where appropriate.

Sections were imaged using a Nikon Eclipse microscope. The calculation of atherosclerotic plaque area and volume was determined using the standard methods, as previously described (10, 11). Plaque area was calculated in three H&E-stained sections (slide sections 1, 10, and 15). The images from these sections were digitized with an Olympus DP80 camera (2001-2005 Olympus Corporation and DP Manager Version 2.2). Plaque area was quantified using the automated region of interest (ROI) tool in ImagePro Plus 9 (Media Cybernetic, Silver Spring, MD). Plaque stenosis was calculated as a percentage of plaque area divided by total vessel area. Plaque area was converted to a volume by averaging the plaque areas in these 3 sections and then multiplied by the total number of slices (*n* = 15).

Plaque elastin and collagen were measured using the ROI tool in ImagePro Plus 9 on the Pentachrome Movat's stain. This was then converted to percentage of plaque area. ImagePro Plus 7 software was also used for immunohistochemical analysis, as previously described (12–14). All analyses were done by investigators who were unaware of the exercise vs. sedentary allocation of the mice from whom the samples were taken.

### Statistical Analyses

The prespecified primary outcome for this study was plaque stenosis. All results are expressed as mean ± SD. Data were assessed for normality using the Shapiro–Wilk test. Data that were not normally distributed were log-transformed for analysis of statistical significance. Statistical comparisons were made by one way ANOVA or two-tailed Student's *t*-test when appropriate using GraphPad Prism version 7.0 (San Diego, CA). Where the ANOVA result was statistically significant, Šídák's multiple comparisons test was conducted.

## Results

A total of one hundred and three mice completed the project. A total of seven mice did not complete the experiment. About one died of an unknown cause and six were removed in accordance with the animal welfare guidelines (two for malocclusion and four due to dermatitis).

### Early-Stage Atherosclerosis Mouse Model

A total of thirty-eight mice completed the early-stage atherosclerosis protocol, 20 in exercise group and 18 controls.

Mice in the exercise group ran 8.13 ± 0.42 km per week. For the sedentary control group, systolic blood pressure (SBP) decreased significantly from baseline over the 10-week intervention period (*p* < 0.01, Table 1). By contrast, SBP increased from baseline in mice who underwent the 10-week exercise intervention (*p* < 0.0001). Diastolic blood pressure (DBP) increased in the control group over the 10 weeks (*p* < 0.001), with no change in the exercise group. For both the control and exercise groups, there were significant increases in body weight from baseline to the end of the 10-week intervention (*p* < 0.01). There were no changes in heart rate for either group.

**Table 1:** The effect of exercise on clinical parameters of the early-stage atherosclerosis mouse model.

**Early-stage atherosclerosis**	**Control (*n =* 18)**		**Exercise (*n =* 20)**	
	**Baseline**	**End**	**Baseline**	**End**
SBP (mmHg)	104 ± 2	101 ± 3^##^	97 ± 7^****^	107 ± 4^***^^####^
DBP (mmHg)	60 ± 1	63 ± 2^###^	70 ± 7^****^	68 ± 4^***^
Heart rate (bpm)	527 ± 41	552 ± 65	569 ± 48	547 ± 51
Body weight (g)	30 ± 2	35 ± 3^####^	29 ± 2	30 ± 2^****^
Heart weight (mg)	-	16 ± 2	-	15 ± 2

*SBP, systolic blood pressure; DBP, diastolic blood pressure; bpm, beats per minute. Data are expressed as mean ± standard deviation. Results are from one-way ANOVA Šídák's post hoc comparison*.

*** *p <0.001 between groups*,

**** *p <0.0001 between groups, (baseline vs. control)*.

## *p <0.01*,

### *p <0.001, and*

##### *p <0.0001 within groups (control vs. exercise at baseline or at end protocol)*.

When directly comparing the exercise and control groups, SBP and DBP were significantly higher in the exercise group than the control group at the end of the intervention (*p* < 0.01, Table 1). There were no differences in body weight between the control and exercise groups at baseline, but at the end of the experimental period, body weights were lower in the exercise group (*p* < 0.01). Furthermore, the percentage increase in body weight over time was significantly higher in the control group than the exercise group (1.6 ± 9.7 vs. 16.9 vs. 7.2%, *p* < 0.0001). No differences were detected in heart rates between groups. However, heart weights measured at the end of the intervention period were greater in the control than the exercise group (*p* < 0.01).

### Comparison Exercise vs. Control Groups: Lipids and Lipoproteins

In the early-stage atherosclerosis mouse model, exercise was associated with lower TC, FC, and TG levels (all *p* < 0.01) (Table 2). Exercise was also associated with lower protein and PL levels (both *p* < 0.001). There was no significant difference in apoB or HDL-C levels between the exercise and control group.

**Table 2:** The effect of exercise on the lipid profile of the early-stage atherosclerosis mouse model.

**Early-stage atherosclerosis**	**Control (*n =* 18)**	**Exercise (*n =* 20)**	***p-* value**
Total cholesterol (mmol/L)	13.9 ± 2.3	11.6 ± 1.8	<0.01
Free cholesterol (mmol/L)	5.9 ± 0.7	5.1 ± 0.7	<0.01
HDL-C (g/L)	0.5 ± 0.2	0.6 ± 0.2	ns
apoB (g/L)^∧^	0.8 ± 0.6	0.7 ± 0.3	ns
Triglycerides (mol/L)	2.9 ± 1.4	1.9 ± 0.9	<0.01
Phospholipid (mmol/L)	6.0 ± 0.9	4.4 ± 0.7	<0.001
Protein (g/L)	34.7 ± 2.5	24.5 ± 3.5	<0.001

*HDL-C and apoB were determined by ELISA. The rest of the results were determined by colorimetric analysis by chemistry analyzer AU480 (Beckman Coulter Inc., CA, USA). Results are from Student's t-tests*.

∧ *data were log-transformed for analysis. p <0.05 is considered significant. HDL-C, high-density lipoprotein cholesterol; apoB, apolipoprotein B*.

### Plaque Volume, Stenosis, and Composition

**Figure 2:**
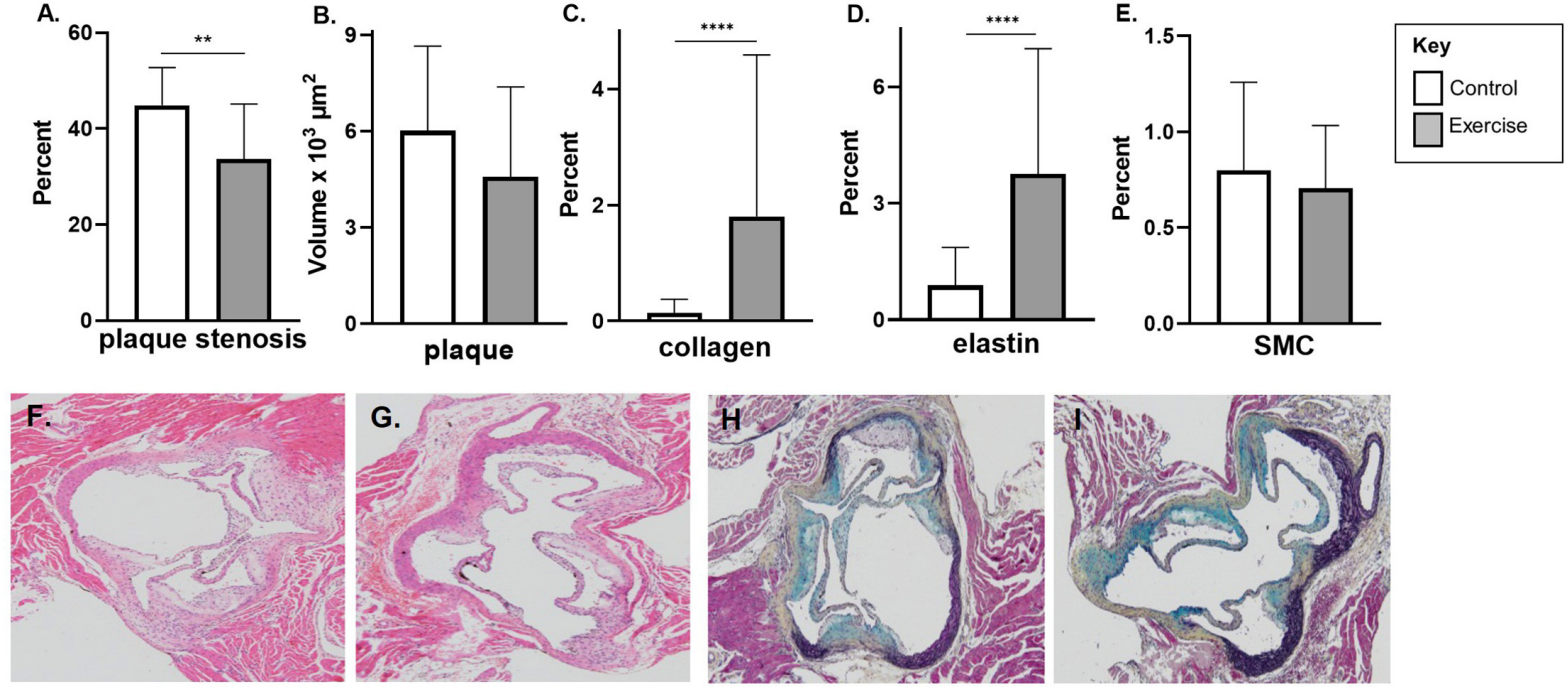
The effect of exercise on plaque volume, degree of stenosis, and composition in an early-stage model of atherosclerosis. Male chow fed *apoE*-/- mice with early-stage plaques were exposed to exercise for 10 weeks at 12 weeks of age. Aortic sinus sections were assessed for **(A)** plaque volume, **(B)** plaque % stenosis, **(C)** vessel elastin, **(D)** plaque collagen and **(E)** plaque SMC content. Data are expressed as mean ± SD. SMC; smooth muscle cell. The *x*-axis represents the control (white bar) vs. the experimental (gray bar). Representative H&E stain of the aortic sinus in the control **(F)** and exercise group **(G)**. Representative H&E stain of the aortic sinus in the control **(F)** and exercise group **(G)**. Representative Movat's stain of the aortic sinus in the control **(H)** and exercise group **(I)** with magnification x 600. **p < 0.01, ****p < 0.0001.

In the early-stage model of atherosclerosis, exercise was associated with significant reduction in percentage plaque stenosis (33.7 ± 11.5% vs. 44.9 ± 7.9%, *p* < 0.01) at the level of the aortic sinus (Figure 2A). There was a non-significant reduction in plaque volume (Figure 2B).

Exercise was associated with higher levels of plaque collagen (1.8 ± 2.8 vs. 0.2 ± 0.2%, *p* < 0.0001) and elastin content (3.8 ± 3.2 vs. 0.9 ± 1.0%, *p* < 0.0001) in the early exercise group (Figures 2C,D). There was no significant difference in SMC percentage between the control and exercise groups (Figure 2E). Representative stained aortic sinus sections for control and exercise groups are shown in Figures 2F,G (H&E) and Figures 2H,I (Movat's).

### Markers of Plaque Stability or Vulnerability

**Figure 3:**
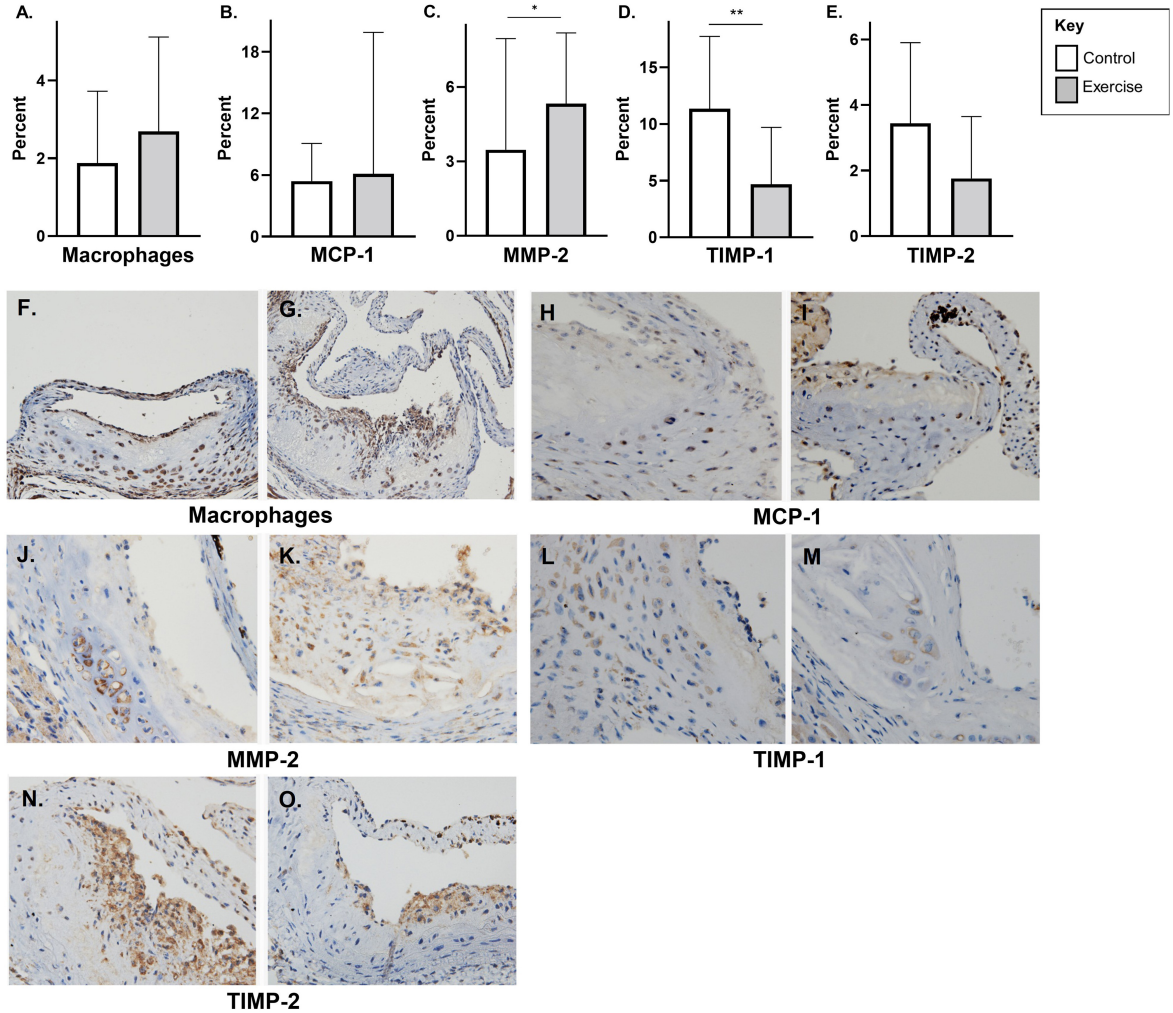
The effect of exercise on F4/80, MMP-2, TIMP, and MCP-1 levels in an early-stage model of atherosclerosis. Chow fed *apoE*-/- mice with early-stage plaques were exposed to exercise for 10 weeks at 12 weeks of age. Aortic sinus sections were assessed for plaque content of **(A)** macrophages (F4/80%), **(B)** monocyte chemoattractant protein-1 % (MCP-1) **(C)** matrix metalloproteinase-2 % (MMP-2), **(D)** tissue inhibitor of metalloproteinase-1 % (TIMP-1), and **(E)** tissue inhibitor of metalloproteinase-2 % (TIMP-2). Data are expressed as mean ± SD. The *x*-axis represents the control (white bar) vs. the exercise group (gray bar). Representative sections of the aortic sinus with stains for macrophage content (F4/80) [**(F)** control, **(G)** exercise], MCP-1 [**(H)** control, **(I)** exercise], MMP-2 [**(J)** control, **(K)** exercise], TIMP-1 [**(L)** control, **(M)** exercise], and TIMP-2 [**(N)** control, **(O)** exercise], with magnification x 600. *p < 0.05 **p < 0.01.

There was no significant difference in macrophage content (F4/80) or MCP-1 with exercise in early-stage plaque (Figures 3A,B). MMP-2 levels were higher with exercise compared to controls in early-stage plaque (3.3 ± 4.5, *p* < 0.05) (Figure 3C). Tissue TIMP-1 was lower with exercise compared to control (4.7 ± 5.0 vs. 11.4 ± 6.4%, *p* < 0.01); however, there was no difference in TIMP-2 levels between the two groups (Figures 3D,E). Representative stained aortic sinus sections for the control and exercise groups are shown in Figures 3F–O.

### Late-Stage Atherosclerosis Mouse Model

A total of sixty-five mice completed the late-stage atherosclerosis protocol; 32 mice were randomized to exercise and 33 to control.

Mice in the exercise intervention ran 6.1 ± 1.4 km per week. In the exercise group, there was an increase in heart rate (*p* < 0.001) and body weight (*p* < 0.0001) over the intervention period (Table 3). There was no change in SBP or DBP in the either the exercise or control group. Body weight also increased in the control group (*p* < 0.001); however, there was no significant difference in heart rate over the intervention period.

**Table 3:** The effect of exercise on clinical parameters of the late-stage atherosclerosis mouse model.

**Late-stage atherosclerosis**	**Control (*n =* 33)**		**Exercise (*n =* 32)**	
	**Baseline**	**End**	**Baseline**	**End**
SBP(mmHg)	100 ± 5	102 ± 4	103 ± 4^*^	102 ± 3
DBP (mmHg)	62 ± 3	61 ± 2	62 ± 2	61 ± 3
Heart rate (bpm)	562 ± 75	562 ± 55	521 ± 64	580 ± 65^##^
Body weight (g)	25 ± 3	38 ± 5^####^	26 ± 2	34 ± 4^****^^####^
Heart weight (mg)	-	19 ± 2	-	19 ± 3

*SBP, systolic blood pressure; DBP, diastolic blood pressure; bpm, beats per minute. Data are expressed as mean ± standard deviation. Results are from one-way ANOVA with Šídák's post hoc comparison*.

* *p <0.05*,

**** *p <0.0001 between groups*.

## *p <0.01*,

#### *p <0.0001 within groups*.

When directly comparing the exercise and control groups at baseline, there was no difference in SBP, DBP, or body weight. Baseline heart rate was however higher in the control group compared to the exercise group (*p* < 0.05) (Table 3). At the end of the intervention period, there was no significant difference in SBP, DBP, heart rate, or heart weight between the control and the exercise group. At the end of the intervention period, body weight was significantly lower in the exercise group compared to control group (*p* < 0.01).

### Comparison Exercise vs. Control Groups: Lipids and Lipoproteins

In the late-stage atherosclerosis group, 10 weeks of exercise was associated with both lower-free cholesterol and phospholipid levels (*p* < 0.05) (Table 4). There was no significant difference in TC, HDL-C, apoB, TG, or total protein levels.

**Table 4:** The effect of exercise on the lipid profile of the late-stage atherosclerosis mouse model.

**Late-stage atherosclerosis**	**Control (*n =* 33)**	**Exercise (*n =* 32)**	***p*-value**
Total cholesterol (mmol/L)	12.7 ± 1.7	13.6 ± 1.9	ns
Free cholesterol (mmol/L)	5.5 ± 0.8	6.0 ± 0.8	<0.05
HDL-C (g/L)	0.4 ± 0.2	0.5 ± 0.3	ns
apoB (g/L) ^∧^	0.5 ± 0.4	0.7 ± 0.4	ns
Triglycerides (mmol/L) ^∧^	1.3 ± 1.0	1.2 ± 0.6	ns
Phospholipid (mmol/L)	4.3 ± 1.0	4.8 ± 0.9	<0.05
Protein (g/L)	32.8 ± 2.7	31.5 ± 2.8	ns

*High-density lipoprotein cholesterol and apoB were determined by ELISA. The rest of the results were determined by colorimetric analysis by chemistry analyzer AU480 (Beckman Coulter Inc., CA, USA). Results are from paired t-tests*.

∧ *data were log-transformed for analysis. HDL-C, high-density lipoprotein cholesterol; apoB, apolipoprotein B. p <0.05 is considered significant*.

### Plaque Volume, Stenosis, and Composition

**Figure 4:**
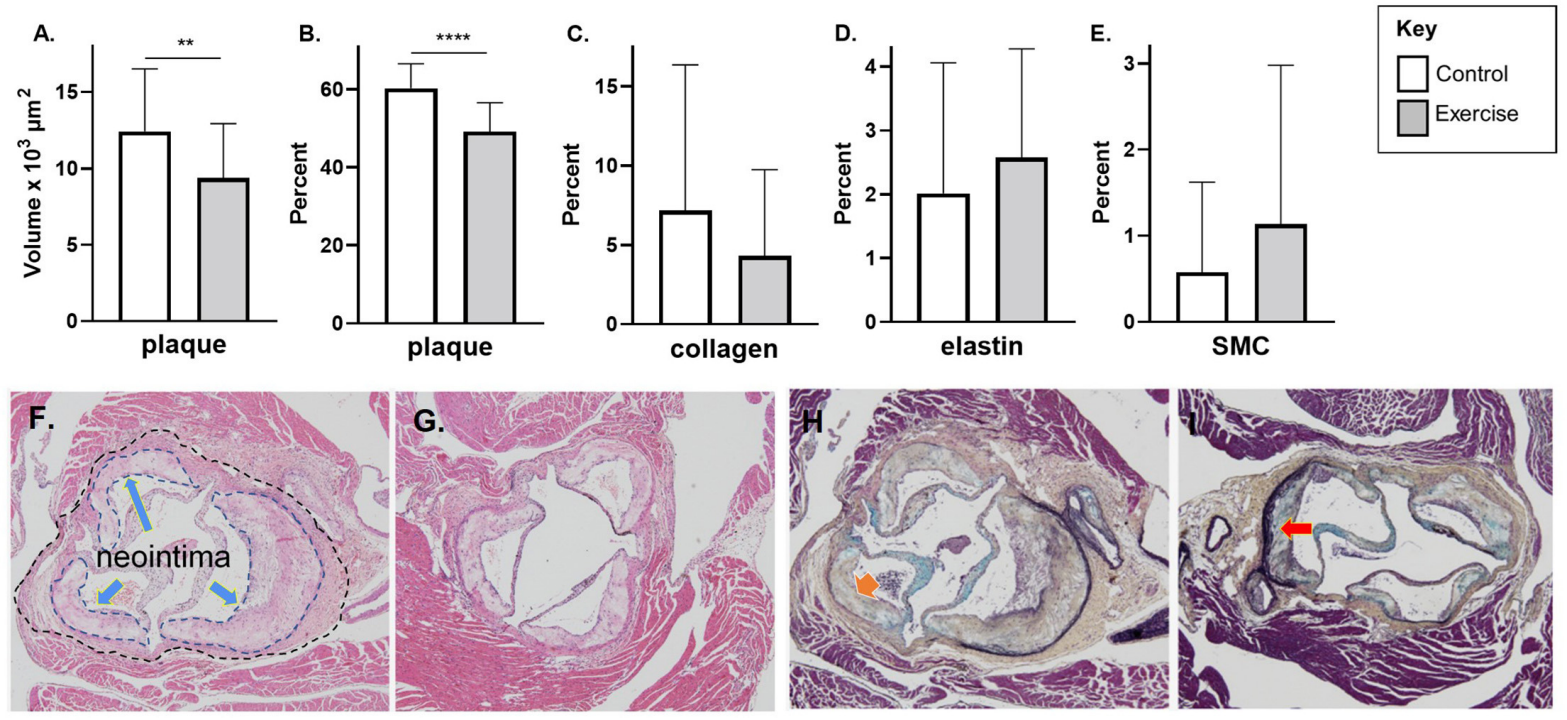
The effect of exercise on plaque volume, degree of stenosis, and composition in a late-stage model of atherosclerosis. Chow fed *apoE*-/- mice with late-stage plaques were exposed to exercise for 10 weeks at 40 weeks of age. Aortic sinus sections were assessed for **(A)** plaque volume, **(B)** plaque % stenosis, **(C)** vessel elastin, **(D)** plaque collagen, and **(E)** plaque SMC content. Data are expressed as mean ± SD. The *x*-axis represents the control (white bar) vs. the experimental (gray bar). Representative H&E stain of the aortic sinus in the control **(F)** and exercise group **(G)** (The inner dotted line in 4F shows the area of plaque formation in this section; the outer dotted lines show the lumen area at the same level of the aortic sinus – refer to Methods for description of plaque volume and stenosis calculations). Representative H&E stain of the aortic sinus in the control **(F)** and exercise group **(G)**. Representative Movat's stain of the aortic sinus in the control **(H)** and exercise group **(I)**. with magnification x 600. Arrows represents collagen **(H)** and elastin **(I)**. *p < 0.05, **p < 0.01. Data are expressed as mean ± SD. SMC, smooth muscle cell. **p < 0.01, ****p < 0.0001.

In the late-stage model of atherosclerosis, exercise was associated with significantly less plaque stenosis (49.2 ± 7.4% vs. 60.3 ± 6.3%, *p* < 0.0001) and plaque volume (9.4 ± 3.6 vs. 12.4 ± 4.1 x10^3^ μm^2^, *p* < 0.01) compared to control (Figures 4A,B). There was no difference in plaque elastin, collagen, or SMC percentage between exercise and control in the late-stage model of atherosclerosis (Figures 4C–E). Representative stained aortic sinus sections for control and exercise groups are shown in Figures 4F,G (H&E) and Figures 4D,H,I (Movat's).

### Markers of Plaque Stability or Vulnerability

**Figure 5:**
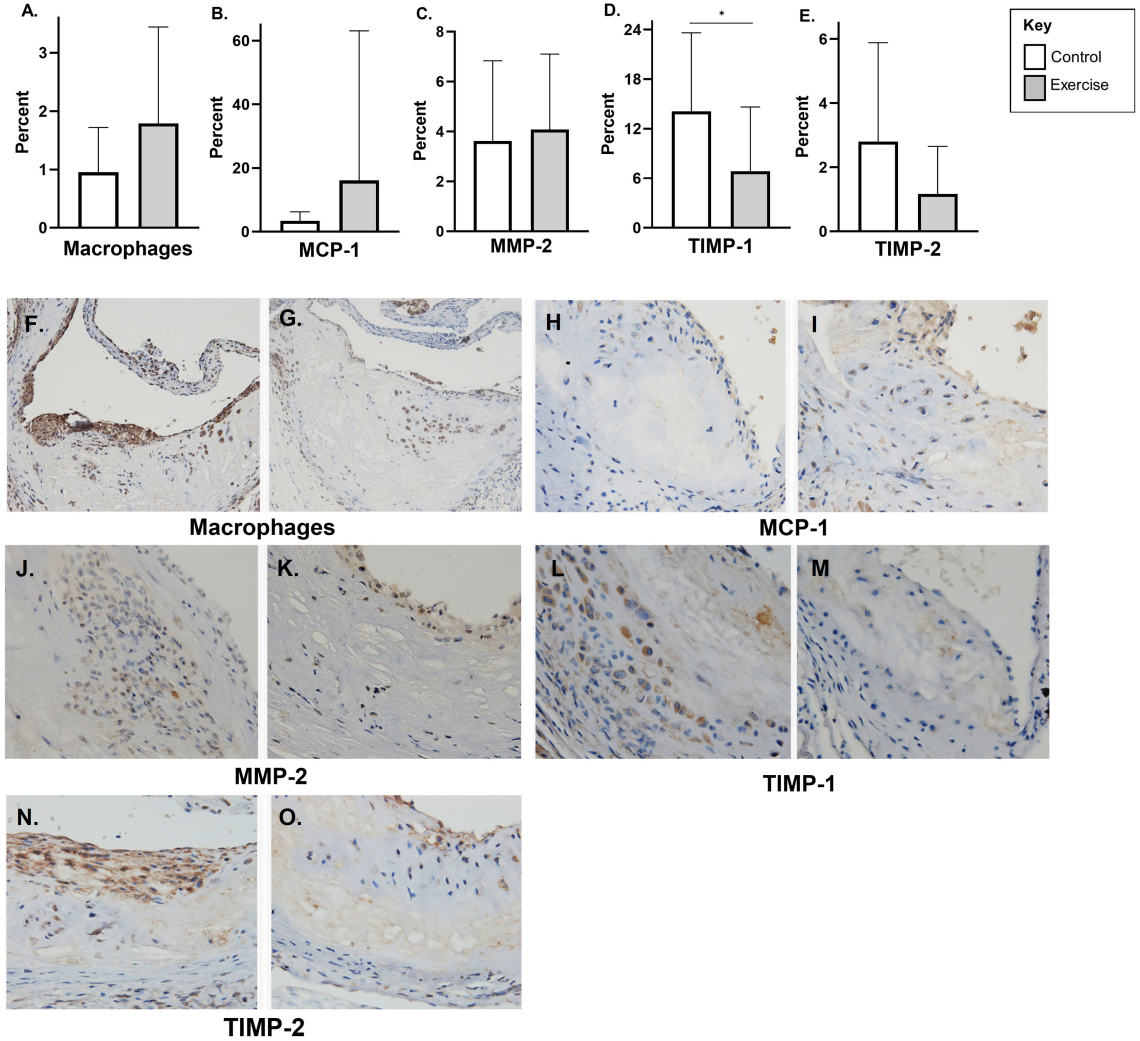
The effect of exercise on F4/80, MMP-2, TIMP, and MCP-1 levels in a late-stage model of atherosclerosis. Chow fed *apoE*-/- mice with late-stage plaques were exposed to exercise for 10 weeks at 40 weeks of age. Aortic sinus sections were assessed for plaque content of **(A)** macrophages (F4/80%), **(B)** monocyte chemoattractant protein-1 % (MCP-1) **(C)** matrix metalloproteinase-2 % (MMP-2), **(D)** tissue inhibitor of metalloproteinase-1 % (TIMP-1), and **(E)** tissue inhibitor of metalloproteinase-2 % (TIMP-2). Data are expressed as mean ± SD. The *x*-axis represents the control (white bar) vs. the exercise group (gray bar). Representative sections of the aortic sinus with stains for macrophage content (F4/80) [**(F)** control, **(G)** exercise], MCP-1 [**(H)** control, **(I)** exercise], MMP-2 [**(J)** control, **(K)** exercise], TIMP-1 [**(L)** control, **(M)** exercise], and TIMP-2 [**(N)** control, **(O)** exercise], with magnification x 600. *p < 0.05.

There was no difference plaque macrophage (F4/80), MCP-1, or MMP-2 in the late-stage atherosclerosis exercise group compared to controls (Figures 5A–C). Tissue TIMP-1 (6.9 ± 7.8% vs. 14.1% ± 9.5 *p* < 0.01) levels were lower with exercise compared to controls; however, there was no difference with TIMP-2 levels (Figures 5D,E). Representative stained aortic sinus sections for the control and exercise groups are shown in Figures 5F–O.

## Discussion

This study showed that exercise reduces plaque stenosis, but it had a differential effect on plaque composition in early-stage compared with late-stage diseases. Exercise had a greater benefit on early-stage atherosclerotic plaque composition, with increased elastin and collagen content compared to controls. Unexpectedly, exercise was associated with a decrease in TIMP-1 levels in both early- and late-stage plaque and higher MMP-2 content in early-stage plaque. There was, however, no significant difference in plaque macrophage, MCP-1, or TIMP-2 content in both early- and late-stage models of atherosclerosis. This study differs substantially from the previous reports, because of the much longer duration of exercise applied, analogous to regular long-term exercise in younger or older adults.

Our data also showed that exercise was associated with an improved lipid profile in early-stage disease. Most notably, early exercise was associated with a lower TC and TG levels whereas there was no difference in HDL-C or apoB levels. The results in the EA group are in contrast to the previous studies, which have found no difference in TC and TG levels; however, of note, most of the studies in the field involve a much shorter exercise intervention period and predominantly involved mice on a high-fat diet (HFD), whereas ours was a chow diet (15, 16).

Exercise decreased atherosclerotic plaque stenosis in both young and aged mice. This is consistent with the previous research that has shown that a 6- or 8-week exercise program in 16-week-old *apoE*-/- mice, fed a HFD, was associated with a decrease in plaque stenosis (6, 16). A similar reduction was found in 8-week-old *apoE*-/- mice undergoing a 6-week swim training program (17). This is in contrast to a study on both young and aged mice where a 5-week exercise intervention did not have any significant effect on % plaque stenosis in an “young” (14 week) or “aged” (49–52 week) mice being fed a chow or a HFD (15). A possible explanation for the discrepant results is that longer periods of exercise (as described here, at 10 weeks) may be required to elicit a significant effect on plaque composition.

This study found that elastin and collagen content were significantly higher in the exercise group but only in the early-stage atherosclerosis model. This finding is consistent with a previous study on *apoE*-/- deficient mice where at 24 weeks of age, a 6-week exercise intervention was associated with increased elastin and collagen content (16). There was no difference in plaque composition with exercise in our late-stage atherosclerosis group, consistent with a previous study which found no difference in plaque content in aged mice (*apoE*-/- 49–52 weeks) fed a chow diet (15). This raises the question as to whether established plaque is more resistant to the beneficial effect of exercise on plaque composition, compared to an earlier stage model of atherosclerosis, or whether there is a differential effect of exercise on “stable” and “unstable” plaques.

Consistent with the previous studies, we found no difference in plaque macrophage or MCP-1 content with exercise in both the early- and late-stage atherosclerosis groups. In contrast to our results, some previous studies have shown that exercise is associated with less plaque macrophage content; however, these studies have been done on younger mice (26 and 30 weeks, compared to 50 weeks in our study) and predominantly in mice exposed to a HFD or other proinflammatory states such as diabetes (16, 18, 19).

The balance between MMP activity and its endogenous inhibitors, TIMPs, is thought to play an important role in atherosclerotic plaque stability, and exercise has been associated with more stable plaque composition (16, 20, 21). As overexpression of TIMPs is postulated to attenuate atherosclerotic plaque development and instability, it was an unexpected finding that TIMP-1 levels were lower with exercise in both the early- and late-stage model of atherosclerosis; however, TIMP-2 levels were unchanged (5, 22, 23). Also unexpected, but in line with decreased TIMP-1 levels, MMP-2 levels were higher in the exercise group in young, but not aged mice. Similar to our finding in aged mice, but in contrast to those found in young mice, a previous exercise study in *apoE*-/- mice found no difference in MMP-2 levels with exercise (16).

There does appear to be a differential effect of individual MMPs and TIMPs on plaque area and composition which needs further exploration. In contrast to our study, a study looking at the effect of exercise on plaque vulnerability in *apoE*-/- mice found that exercise alone decreased TIMP-2 levels and increased TIMP-1 levels (6). Increased TIMP-1 levels have also been reported after a 6-week exercise program in *apoE*-/- mice fed a HFD, there was no difference in MMP-2 levels, and however, MMP-2 levels decreased with exercise (16). The previous research has also shown that TIMP-2/*apoE* knock and MMP-2 /*apoE* knockout mice but not TIMP-1/*apoE* knockout mice had more complex and vulnerable plaques after being fed a HFD compared to *apoE* knockout controls (24, 25). This supports a differential effect of TIMP-1 and 2 s on plaque development and stability suggests that TIMP-2 may play a more prominent role in plaque stability. This is an important area for future investigation.

### Strengths

This is one of the first studies to assess an exercise intervention in both an early- and late-stage model of atherosclerosis. Furthermore, the exercise intervention was longer than most exercise interventions in the literature (at 10 weeks), which allows a better assessment on the effect of long-term exercise on atherosclerosis. The sample size was also larger than the most other previous studies of this type.

### Limitations

Although diet was controlled, mice were able to eat *ad libitum;* hence, the exercising mice are likely to have consumed more calories. There were some baseline differences in blood pressure between the control and exercise group which may have had a (likely minor) impact on plaque progression. It is difficult to directly compare to many studies in the field, as most studies used a HFD, in *apoE*-/- mice.

Another limitation of this study is that although the duration of exercise intervention was the same in the early- and late-stage atherosclerosis groups, the amount of physical activity was different between these groups. The age of the mice may also contribute to the differences observed, rather than just the stage of disease studied.

Finally, we did not assess the corresponding mRNAs which would offer more information to support our conclusions concerning plaque composition. This is a potential area for future research.

## Conclusions

This study shows that exercise has a beneficial role in reducing plaque stenosis in an animal model of both early- and late-stage atherosclerosis. There were differential effects of exercise on early- and late-stage plaque composition and also plasma lipid profile, which suggest that exercise had a greater impact on early-stage plaque compared with late-stage plaque. An unexpected finding was that exercise was associated with increased TIMP-1 but not TIMP-2 levels in both young and aged mice. MMP-2 levels were also higher with exercise in young mice. This differential impact of exercise highlights that the interventions aimed at early-stage atherosclerotic disease may be more effective at influencing traditional measures of atherosclerosis (plaque stenosis and composition); however, the effect of exercise on the complex interplay between MMPs and TIMPs in both early- and late-stage plaque may be an important area for future investigations.

## Data Availability Statement

The data that support the findings of this study are available from the corresponding author, upon reasonable request.

## Ethics Statement

The animal study was reviewed and approved by the Royal Prince Alfred Hospital Animal Ethics Committee (protocol number 2014-011).

## Author Contributions

KS, CB, and DC contributed to conception and design of the study. KS, VK, HL, SB, CB, and DC contributed to the methodology. KS, HL, and VK performed the investigations. Formal analysis was conducted by KS, HL, and SB. KS wrote the first draft of the manuscript. All authors contributed to the manuscript revision and approved the submitted version.

## Funding

This work was supported by the National Health and Medical Research Council of Australia Program Grant APP1037903 to DC. KS was supported by a National Health and Medical Research Council of Australia postgraduate scholarship.

## Conflict of Interest

The authors declare that the research was conducted in the absence of any commercial or financial relationships that could be construed as a potential conflict of interest.

## Publisher's Note

All claims expressed in this article are solely those of the authors and do not necessarily represent those of their affiliated organizations, or those of the publisher, the editors and the reviewers. Any product that may be evaluated in this article, or claim that may be made by its manufacturer, is not guaranteed or endorsed by the publisher.
